# Adenovirus-vectored African Swine Fever Virus Antigens Cocktail Is Not Protective against Virulent Arm07 Isolate in Eurasian Wild Boar

**DOI:** 10.3390/pathogens9030171

**Published:** 2020-02-28

**Authors:** Estefanía Cadenas-Fernández, Jose M. Sánchez-Vizcaíno, Aleksandra Kosowska, Belén Rivera, Francisco Mayoral-Alegre, Antonio Rodríguez-Bertos, Jianxiu Yao, Jocelyn Bray, Shehnaz Lokhandwala, Waithaka Mwangi, Jose A. Barasona

**Affiliations:** 1 VISAVET Health Surveillance Centre, Complutense University of Madrid, 28040 Madrid, Spain; jmsanche@ucm.es (J.M.S.-V.); alkosows@ucm.es (A.K.); belenriv@ucm.es (B.R.); fjmayoral@ucm.es (F.M.-A.); arbertos@visavet.ucm.es (A.R.-B.); jbarason@ucm.es (J.A.B.); 2 Department of Animal Health, Faculty of Veterinary, Complutense University of Madrid, 28040 Madrid, Spain; 3 Department of Animal Medicine and Surgery, Faculty of Veterinary, Complutense University of Madrid, 28040 Madrid, Spain; 4 Department of Diagnostic Medicine/Pathobiology, Kansas State University, Manhattan, KS 66506, USA; jianxiuy@ksu.edu (J.Y.); shehnaz@vet.k-state.edu (S.L.); 5 Department of Veterinary Pathobiology, Texas A&M University, College Station Texas, TX 77843-4467, USA; jmbray@cvm.tamu.edu

**Keywords:** African swine fever, adenovirus, Armenia07/Arm07, Eurasian wild boar, immune response, subunit vaccine, protective efficacy

## Abstract

African swine fever (ASF) is a viral disease of domestic and wild suids for which there is currently no vaccine or treatment available. The recent spread of ASF virus (ASFV) through Europe and Asia is causing enormous economic and animal losses. Unfortunately, the measures taken so far are insufficient and an effective vaccine against ASFV needs to be urgently developed. We hypothesized that immunization with a cocktail of thirty-five rationally selected antigens would improve the protective efficacy of subunit vaccine prototypes given that the combination of fewer immunogenic antigens (between 2 and 22) has failed to elicit protective efficacy. To this end, immunogenicity and efficacy of thirty-five adenovirus-vectored ASFV antigens were evaluated in wild boar. The treated animals were divided into different groups to test the use of BioMize adjuvant and different inoculation strategies. Forty-eight days after priming, the nine treated and two control wild boar were challenged with the virulent ASFV Arm07 isolate. All animals showed clinical signs and pathological findings consistent with ASF. This lack of protection is in line with other studies with subunit vaccine prototypes, demonstrating that there is still much room for improvement to obtain an effective subunit ASFV vaccine.

## 1. Introduction

African swine fever (ASF) is a viral disease of domestic and wild suids for which there is currently no vaccine or treatment available. ASF causes a spectrum of disease manifestation, from highly lethal to subclinical, depending on the immunological characteristics of the host and the virulence of the virus [1,2]. The ASFV isolates spreading through Europe since 2007 and Asia since 2018 [3] are highly virulent, leading to high lethality rates of up to 95–100% [4,5]. Counter-measures for controlling the spread of current ASFV outbreaks and eradication are based on rapid virus detection and establishment of strict sanitary measures, including animal stamping out and regional, national, and international trade restrictions [1]. These result in important direct costs and export losses leading to a great socioeconomic impact in affected regions.

Furthermore, these control measures are insufficient where wild boar are widely affected [6] such as in the European Union, where wild boar represents more than 90% of the outbreaks reported [3], and the outbreaks in domestic pigs are sporadic and related to the presence of ASFV in wild boar [7]. Thus, new control strategies are urgently needed. Vaccine development is considered as the major gap in ASF control and eradication [8], with a particular interest in vaccines for wild boar given the importance of vaccinating wild boar, as demonstrated with classical swine fever [9]. 

Several factors hinder the development of the ASF vaccine, including the complexity of ASFV, lack of characterization of virus–host interactions and mechanisms of protective immunity. Some studies have demonstrated that immunization with inactivated ASFV or live-vectored subunit vaccines can induce antibody responses, but they do not confer strong protection [10]. Attenuated vaccines have demonstrated the potential to protect domestic pigs and wild boar against experimental infection with the virulent virus [11,12]. The use of this type of vaccine has some limitations such as safety concerns related to their inherent infectious nature [11]. Attenuated or virulent virus isolates can be modified genetically to be less virulent and safer [11,13,14,15,16], but the deletion of virulence-associated genes could reduce the ability of the viruses to confer protection against the virulent isolate. However, it must be noted that some of these mutants have shown a good degree of protection (60–100%) against challenge with the virulent isolate Armenia 2007 [11]. 

Subunit vaccines based on some of the most extensively studied ASFV antigens, such as p32, p54, and p72 envelope protein, have been tested as vaccine candidates either as virus-expressed recombinant proteins [17,18,19] or via DNA plasmid delivery [20]. Studies suggest that these antigens play a relevant role in protection, but are not capable of conferring full protection when they are used singly or in combination [21]. However, subunit vaccines have the advantage of being safe in contrast to attenuated vaccines, and, therefore, there is great interest in continuing the development and validation of this type of vaccine.

We hypothesized that immunization with a cocktail of thirty-five rationally selected antigens would improve protective efficacy given that the correlates of protection are poorly defined, protective antigen(s) are not known, and combinations of immunogenic antigens tested so far have failed to elicit protective efficacy similar to attenuated vaccines [18,19,20,21,22]. Previous studies have shown that immunizing domestic pigs using a cocktail of replication-deficient adenoviruses expressing ASFV antigens p32, p54, pp62, and p72 or A151R, B119L, B602L, EP402RΔPRR, B438L, K205R-A104R, pp62, p72, and pp220 elicited robust antigen-specific antibody, IFN-γ cellular, and cytotoxic T-lymphocyte (CTL) responses [17,18,19]. In this study, we tested a prototype vaccine containing thirty-five adenovirus-vectored ASFV antigens in wild boar to evaluate their protective efficacy against the virulent ASFV Arm07 isolate. The cocktail contained adenoviruses expressing the above named antigens and others such as EP153R, p10, p15, CP80R, I329L, H108R, K196R, CP312R, F334L, NP419L, NP868R, B66L, H339R, and R298L, which have previously been shown to be immunogenic in domestic pigs [20,21,22,23,24,25]. The rest (K145R, B385R, F165R, F778R, S273R, MGF100-1L, A224L, MGF505-6R, and B175L)were selected based on the presence of putative T cell epitopes as judged by the presence of predicted strong binding peptides to multiple well-characterized *SLA-I* alleles [NetMHCpan4.0].

## 2. Materials and Methods

### 2.1. Animals

Animal experiments were performed under biosafety level 3 conditions at the facilities of the VISAVET Centre at the University Complutense of Madrid. For the experiment, 13 female wild boar piglets, 3–4 months old and weighing 10–15 kg, were obtained from a commercial wild boar farm in Extremadura, Spain. These piglets had not been vaccinated against any infectious disease and had tested negative for the following main porcine pathogens in the region: Aujeszky virus, *Mycobacterium bovis*, *Mycoplasma pneumoniae*, and porcine circovirus type 2. The animals were acclimated for two weeks before experiments began. Access to water and food was provided ad libitum throughout the study. Animal care and procedures were performed in accordance with the guidelines of the good experimental practices, following European, national, and regional regulations and under the supervision and approval of the Ethics Committee of Comunidad de Madrid (reference PROEX 004/18). The approved protocol included a detailed description of efforts to avoid and prevent unnecessary suffering of the animals, including humane endpoints and guidelines to follow for euthanasia.

### 2.2. Generation of Recombinant Adenoviruses Expressing ASFV Antigens

**Figure 1:**
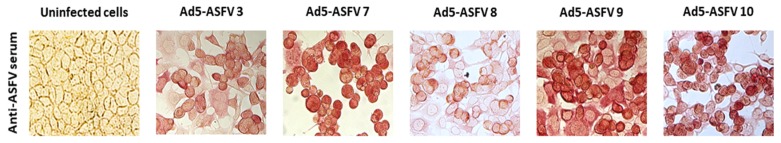
Protein expression by Ad5-ASFV constructs. The expression and authenticity of the ASFV antigens encoded by the generated recombinant adenoviruses were evaluated by immunocytometric analysis of Ad5-ASFV-infected HEK-293A cells probed with anti-FLAG monoclonal antibody [data not shown] and then validated by using ASFV-specific convalescent serum. Uninfected HEK-293A cells served as negative controls. Protein expression by representative constructs is shown.

The recombinant adenoviruses expressing the selected ASFV antigens, as well as the negative control Ad-Luc (Ad-Luciferase), were generated, scaled up, and titrated as previously described (infectious focus units [IFU]) [17,18,19]. Thirty-five antigens were used to generate adenovirus expression constructs. Due to its large size, the pp220 polyprotein (Georgia 2007/1 isolate; GenBank Accession FR682468) sequence was split into two parts designated p220.1 and p220.2 (Table 1). Briefly, the polypeptide sequences of the ASFV antigens were modified to add in-frame, HA, and FLAG tags at their N- and C-termini, respectively. The modified protein sequences were used to generate synthetic genes (GenScript) which were codon-optimized for expression in swine. All the genes were then used to generate recombinant replication-incompetent adenoviruses encoding the ASFV genes (designated Ad5-ASFV 1 to 10) using the ViraPower™ Adenoviral Gateway™ Expression Kit (Thermo Fisher Scientific K493000). Antigen expression by the adenoviruses was confirmed as previously described [17,18,19] (Figure 1). 

### 2.3. ASFV for Challenge

The highly virulent ASFV genotype II Arm07 isolate was used for the challenge [11,12]. The Arm07 isolate was obtained from the European Union Reference Laboratory for ASF, Centro de Investigación en Sanidad Animal, Instituto Nacional de Tecnología Agraria y Alimentaria (CISA-INIA). This virus was propagated in porcine blood monocytes as previously described [11]. The viral titer was defined as the amount of virus causing hemadsorption in 50% of infected cultures (HAD_50_) per milliliter.

### 2.4. Experimental Design: Immunization and Challenge

The treated animals were divided into three different groups: the first group of wild boar was inoculated with the Ad5-ASFV cocktail formulated in BioMize adjuvant [VaxLiant] under prime–boost strategy (T1; n = 4); the second group was inoculated with the Ad5-ASFV cocktail formulated in adjuvant BioMize under single-dose strategy (T2; n = 4); and one wild boar was inoculated with the Ad5-ASFV cocktail but with no adjuvant under prime–boost immunization regimen (T3; n = 1). The controls received a prime and boost dose of the Ad5-Luc formulated in BioMize adjuvant (Control; n = 2). Finally, another group was inoculated intramuscularly (IM) with a highly virulent ASFV isolate, Arm07, at the time of the challenge in order to perform a shedder-animal challenge-exposure infection model (Challenge; n = 2) (Table 2).

Thirty-five days post-priming (dpp) (10^10^ IFU), animals that were boosted received the second dose (10^11^ IFU) of their corresponding recombinant formulation. The inoculums (4 mL) were administered IM in the neck region behind the ears. From the inoculation of the prime dose (0 dpp) until the inoculation of challenge (47 dpp; 0 days post-challenge, dpc) is hereby named as the immunization period.

During the immunization period, blood for the preparation of serum was collected once a week from all the animals. The serum samples were tested to detect ASFV-specific antibodies using a commercial ELISA test based on the detection of p72 ASFV antigen (Ingenasa-Ingezim PPA Compac K3; Ingenasa, Madrid, Spain) according to the manufacturer’s instructions (sensitivity: 98%, specificity: 100%).

Forty-eight days post-priming, a shedder-pig challenge-exposure infection model was used to evaluate the protective efficacy of the Ad5-ASFV cocktail. To this end, two naïve wild boar were IM challenged with 10 HAD_50_ of ASFV Arm07 and, at the same time, they were exposed to the nine treated and two control animals. From this time until the end of the trial is hereby named as the challenge period. 

During the challenge period, EDTA-blood and coagulated blood for the preparation of serum were collected from each animal twice a week. To detect ASFV DNA in blood (viremia), a quantitative PCR assay was used [26]. Serum samples were tested to detect specific antibodies against ASFV, as mentioned above.

### 2.5. Clinical Sign Monitoring

The animals were observed daily throughout the trial to monitor their health status, by a video camera (recording 24 hours a day) and visits by a wildlife-specialist veterinarian. In addition, prior to sampling, rectal temperature was measured once a week during the immunization period and twice a week during the challenge period, as well as in animals showing any clinical sign. Fever was defined as a rectal temperature greater than or equal to 40 °C.

The evolution of the disease was expressed in terms of a quantitative clinical score (CS) specific for ASFV infection in wild boar (see Table 3). The CS was stablished following Gallardo et al. (2017) and Galindo-Cardiel et al. (2013) clinical evaluation guidelines for domestic pigs [27,28], but with slight modifications established by four wildlife-specialist veterinarians based on previous and current studies [12], in order to obtain a more accurate and sensitive clinical observation of ASFV infection course in wild boar. This CS considers nine parameters (rectal temperature, behavior, body condition, skin alterations, ocular/nasal discharge, joint welling, respiratory symptoms, digestive symptoms, and neurological symptoms), the degree of severity of which is measured from 0 to 4 (most severe). All clinical observations were daily recorded, except temperature in order to minimize animal handling and stress.

The recorded CS was used to define humane endpoints. In this sense, euthanasia was performed following humane endpoints described by Gallardo et al. [29] if the accumulative CS was >18, or animals showed any of the following severe clinical signs (level 4) for more than two consecutive days: fever, anorexia, recumbency, respiratory or digestive symptoms. Animals that were unacceptably suffering without reaching the endpoint, based on veterinary criteria, were also euthanized.

### 2.6. Necropsy and Sample Collection of Tissues

The experiment lasted until 15 dpc (62 dpp). Animals which reached the pre-defined humane endpoint or were unacceptably suffering based on veterinary criteria were euthanized via intravenous injection of T61® (Intervet, Spain) following anesthesia by intramuscular injection of combination tiletamine-zolazepam (Zoletil 100 mg/mL, Virbac, France, target dose 3 mg/kg) and medetomidine (Medetor, Virbac, France, target dose 0.05 mg/kg) [30]. Necropsy was performed on all animals [wild boar treated (T1, T2, and T3), control, and IM challenge groups] to confirm or exclude the presence of pathological lesions consistent with ASF. Tissue samples of spleen, kidneys, liver, brain, bone marrow, pool of first barrier organs/peripheral lymph nodes (tonsils, submandibular, retropharyngeal, prescapular, and inguinal), abdominal cavity organs (intestine, urinary bladder, renal lymph node, gastrohepatic lymph node, and mesenteric lymph node), and thoracic cavity organs (heart, lung, and mediastinal lymph node) were collected from each necropsied animal for detection of ASFV DNA using a quantitative PCR assay [26].

### 2.7. Statistical Analysis

The statistical analyses were conducted using R 3.5.0 and SPSS 20 (IBM, Somar, NY, USA) [31]. A *p*-value < 0.05 was considered statistically significant.

Kaplan–Meier survival curve and Mantel–Cox log-rank test were used, respectively, to compute the probability of death and to test for significant survival differences among treated T1, T2, and T3 and control groups. Kruskal–Wallis test was used to compare the time of clinical onset of the disease, the clinical score, the temperature, the CT values of blood samples and the start time of viremia among the different treatment groups and the controls. One-way ANOVA was used to compare the cycle threshold (CT) values from quantitative PCR among tissues tested.

A generalized mixed-effects model was generated to evaluate which factors shape the ASFV DNA levels in tissues. The CT value from each tissue sample and animal was used as the response variable. To control individual correlation, a variable included ID (individual identification of each wild boar) was used as a random factor. The following were considered as the explicative variables: tissue tested, group of animals, survival time in days, viremia duration in days, CT value of the last viremia, last CS, last rectal temperature, and last ELISA result (positive and negative). For the model analyses, data exploration followed by a backward stepwise model selection based on Akaike information criteria was performed [32]. The Bayesian information criterion [33] was also taken into account for obtaining the most parsimonious model. The goodness of fit was assessed by calculating the pseudo R squared for generalized mixed-effect models according to Nakagawa and Schielzeth [34]. The conditional R^2^ refers to the proportion of variation explained by both fixed and random factors, and the marginal R^2^ describes the proportion of variation explained only by fixed factors.

## 3. Results

### 3.1. Outcomes during the Immunization Period

Neither local nor systematic adverse side-effects were observed in the treated and control animals during the immunization period. Prior to the challenge, only one of the nine treated animals, which belonged to the T1 group, was positive for anti-ASFV antibodies at 12 dpp, although this antibody response was temporary and it did not remain in the next sampling. 

### 3.2. Outcomes during the Challenge Period

**Figure 2:**
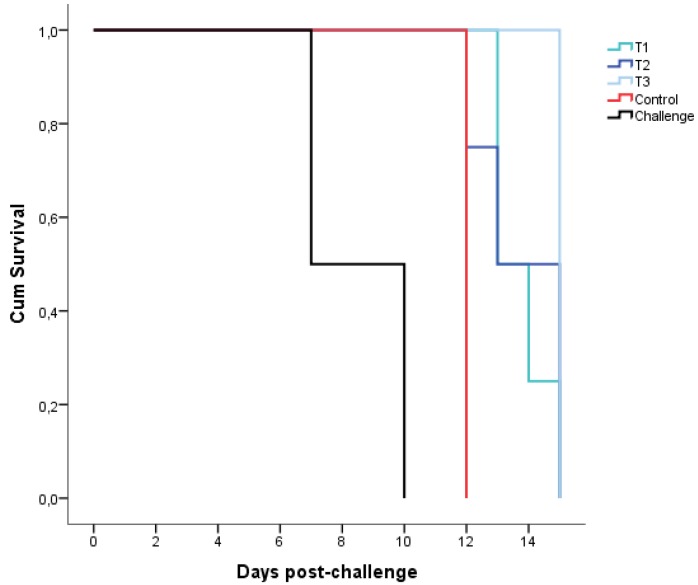
Curve of Kaplan–Meier showing the data of the survival time of the treated (T1, n = 4, green; T2, n = 4, dark blue; T3, n = 1, light blue), the control (n = 2, red), and the intramuscularly challenged wild boar (n = 2, black).

The two IM challenged animals died at 7 and 10 dpc, respectively. The two control animals died at 12 dpc. None of the nine treated animals were protected against the highly virulent ASFV Arm07 isolate; all animals died or were euthanized, based on the humane endpoint described above, at 14 ± 1 dpc (T1), 14 ± 2 dpc (T2), and 15 dpc (T3). There were no significant differences among the treatment groups and the controls on survival time (Mantel–Cox, χ2 = 6.804, 3 d.f.; *p* = 0.078) (Figure 2). 

After the challenge by contact with the two animals that received ASFV IM inoculation, three out of the nine treated animals were positive for anti-ASFV antibodies. Two of these treated animals (from T2 and T3 groups, respectively) had ASFV-specific antibodies in the last sampling before succumbing to the disease, which was 14 dpc. Another animal (from T1 group) had ASFV-specific antibodies in sera from blood collected in its last two consecutive samplings, 11 and 13 dpc. 

**Figure 3:**
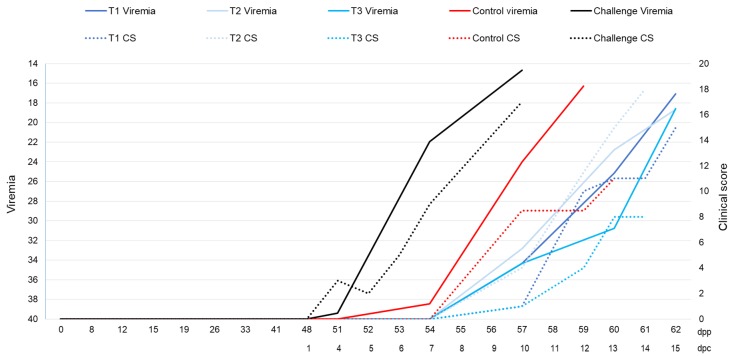
The average clinical score (CS) and the cycle threshold (CT) determined by quantitative PCR of the treated (T1, n = 4; T2, n = 4; T3, n = 1; blue), control (n = 2, red), and IM challenged wild boar (n = 2, black). Averages are shown at different days post-priming (dpp), including days post-challenge (dpc). The CS displayed corresponds to sampling days where temperature was measured.

All the treated and control animals showed clinical signs consistent with ASF from 10 ± 3 dpc, mainly high fever, partial lethargy, and slight anorexia. Some animals also showed slight erythema, nasal and ocular discharges, mucus in feces, slight walking difficulties, increased respiratory rate, and coughing. Overall, the IM challenged animals showed clinical onset of the disease (CS ≥ 8) from 6 and 7 dpc, only one of the two control animals reached a CS ≥ 8 from 10 dpc, and the treated animals from T1, T2 and T3 groups showed clinical onset of the disease from 12 to 13 dpc (Figure 3). However, no significant differences were observed among the treatment groups and the controls regarding the time of clinical onset, the clinical score, and the temperature (Kruskal–Wallis test, *p* = 0.195; *p* = 0.922; *p* = 0.831, respectively).

The IM challenged animals showed viremia 3 and 7 dpc with CT = 38.80 and 21.72, respectively, until death (CT = 22.20 and 14.68, respectively). The control animals showed viremia 7 and 10 dpc with CT = 38.46 and 18.62, respectively, which also was maintained until death (CT = 17.14 and 15.47, respectively). The treated animals started to show viremia 11 ± 2 dpc (T1, CT = 28.58 ± 7.89; T2, CT = 28.89 ± 4.96; T3, CT = 34.34), until death (T1, CT = 19.41 ± 3.47; T2, CT = 17.71 ± 1.18; T3, CT = 18.59) (Figure 3). The start time of the viremia and its CT value were not significantly different among the treatment groups and the controls (Kruskal–Wallis test, *p* = 0.401; *p* = 0.794).

### 3.3. Outcomes of Necropsy and Tissues Tested

Post-mortem analyses revealed pathological lesions consistent with ASF in all the treatment and control animals. The main necropsy findings were congestion and focal hemorrhages on the lung surface, spleen (splenomegaly), lymph nodes (lymphadenomegaly), kidneys, liver (hepatomegaly), and intestine mucosa. In addition, moderate to severe accumulation of yellowish to reddish fluid was frequently observed in the abdominal cavity (ascites), thorax (hydrothorax), and pericardial sac (hydropericardium). 

**Figure 4:**
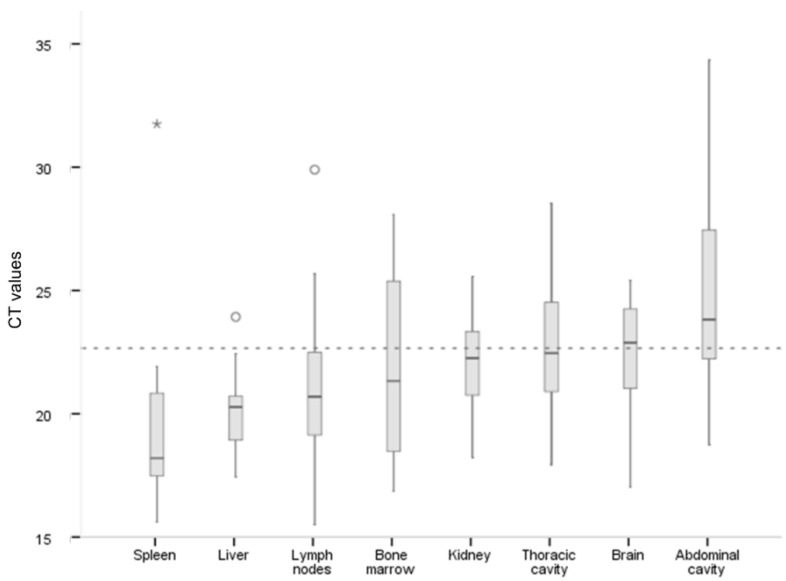
The average of ASFV DNA levels (expressed as the cycle threshold [CT] from quantitative PCR) in post-mortem tissues from all wild boar, treated and controls (n = 13).

In general, the averages of ASFV DNA levels obtained from spleen (CT = 19.86 ± 4.04), liver (CT = 20.09 ± 1.84), first barrier organs/peripheral lymph nodes (CT = 20.97 ± 2.50), and bone marrow (CT = 21.83 ± 3.82) were significantly higher compared with ASFV DNA levels obtained from tissues of abdominal cavity (CT = 24.83 ± 3.44) (one-way ANOVA, *F* = 10.74, *p* < 0.05). The averages of ASFV DNA levels obtained from spleen and first barrier organs/peripheral lymph nodes were significantly higher than those obtained from tissues of thoracic cavity (CT = 23.10 ± 2.85) (one-way ANOVA, *F* = 10.74, *p* < 0.05) (Figure 4).

The best-fitting model parameterized to identify factors explaining the ASFV DNA levels in tissues included the following predictor variables: tissue tested, viremia duration in days, last CS, and last ELISA test result (Table S1). 

The results obtained by the best-fitting model showed that the ASFV DNA levels in tissues were lower in abdominal cavity organs than the rest of the tissues tested (bone marrow, brain, kidneys, liver, first barrier organs/peripheral lymph nodes, spleen, and thoracic cavity organs) (Table 4 and Figure S2). In addition, this model indicates that higher ASFV DNA levels were negatively associated with CS; animals with more clinical signs showed lower ASFV DNA levels in tissues. However, higher ASFV DNA levels were positively associated with days of viremia; in other words, animals with longer viremia showed higher ASFV DNA levels in tissues. Finally, animals positive for anti-ASFV antibodies showed lower ASFV DNA levels in tissues.

## 4. Discussion

The thirty-five Ad5-ASFV antigens cocktail was tested for protective efficacy in wild boar. No specific maintained antibodies against ASFV were detectable by ELISA test in any of the treated animals prior to the challenge. This outcome was inconsistent with the robust primary and recall antibody responses that were observed in domestic pigs with four and seven adenovirus-vectored ASFV antigens [17,18]. Upon challenge with virulent ASFV Arm07 isolate, the treated animals were unprotected and developed ASF disease similar to the controls. However, after the challenge, three treated animals displayed a positive specific antibody response against the p72 ASFV antigen. 

The lack of protection observed in this study with an inoculation of thirty-five Ad5-ASFV antigens cocktail, including novel antigens associated with high immune response and a modified CD2v relevant for protection [19,35,36,37,38], is in line with previous outcomes from domestic pigs immunized with nine and six Ad5-ASFV antigens cocktails [19]. This suggests the increase in the number of ASFV antigens did not improve protection. On the other hand, the treated animals in this study (T1, T2, and T3) did not show an enhancement of the disease in contrast with the previous trial conducted in domestic pigs with nine and six Ad5-ASFV antigens cocktails [19]. Outcome differences between these two studies may be due to differences in the antigens selected and the experimental design, such as the animal host (domestic pigs/wild boar) and the ASFV challenge isolate (Georgia 2007/Arm07). 

The lack of protection is consistent among studies that have evaluated subunit vaccine prototypes [19,20,36,37,38,39,40,41], which is inconsistent with protective efficacy obtained with live attenuated ASFV vaccine prototypes [11,12,13,14,16,42,43,44,45]. The failure of subunit vaccine prototypes to confer protection could indicate that the specific combination of ASFV antigens to induce a protective immune response is yet to be determined. The fact that none of the viral proteins have demonstrated sufficient induction of robust protective immunity to date, and the presence of numerous discrepancies on the role in protective immunity among the ASFV antigens previously described [10,11,20,21,33,34,35,36], emphasizes the need for further studies on evaluating protective efficacy of novel ASFV antigens in order to identify key protective antigens [21].

On the other hand, these failures could also indicate that in vivo antigen delivery by the prototype subunit vaccines is ineffective at eliciting appropriate immune responses required for protection. Thus, the definition of the correlates of protection, improving immunization strategies including expression and post-translational modification of the ASFV antigens, and optimal antigen dose and administration route [10] could be important to enhance subunit vaccine efficacy. One strategy to enhance the protective efficacy of the current vaccine prototype could be using heterologous prime–boost immunization, such as priming with adenovirus followed by heterologous boosting using a live vector backbone. This combination has been shown to enhance both antibody and T cell responses in other species [46,47]. Furthermore, a recent study in pigs that used such a heterologous prime–boost strategy expressing ASFV antigens showed a significant increase in antibody response induced by MVA boost; however, it did not increase the cellular response [21]. Thus, further delivery/vector systems and immunization strategies need to be developed and evaluated. 

No significant differences among the treatment groups (T1, T2, and T3) were observed regarding immune response or protection. However, it is difficult to say there was no difference between prime–boost and single-dose immunization strategies or whether the use of the BioMize adjuvant had any effect given the low sampling number in each group. Nevertheless, the fact that the lowest CS recorded after the challenge was in the treated animal with no adjuvant is consistent with the previous study [19], where disease enhancement was observed in the treated animals with the BioMize adjuvant compared with the ZTS-01 adjuvant. In this sense, based on previous and current outcomes, the BioMize adjuvant did not improve the efficacy of this vaccine construct. This idea is related to the lack of enhancement of efficacy through the use of modern adjuvants with inactivated ASFV vaccine observed previously [48]. 

The data obtained in the current study enabled us to perform a statistical model analysis to describe some interesting facts related to the ASFV DNA levels in tissues that have not yet been thoroughly investigated and we recommend further studies to support our results. In this way, spleen and liver were detected as the tissues with higher ASFV DNA levels. Thus, spleen and liver can be established as the target tissues in order to detect the presence of ASFV DNA. In contrast, other tissues from the abdominal cavity (intestine, urinary bladder, renal lymph node, gastrohepatic lymph node, and mesenteric lymph node) showed the lowest ASFV DNA levels compared to all the remaining tissues tested (see Figure S2). The high ASFV DNA level in spleen compared with other tissues has been seen in previous studies [4,49], while the liver has rarely been tested to detect ASFV DNA.

Interestingly, higher CS was related to lower ASFV DNA levels in tissues. This could be explained by the peracute ASF clinical form developed by some animals, characterized by high ASFV DNA level in tissues but the absence of clinical signs [1] because they do not have enough time to display the clinical disease. In addition, animals with positive antibody response showed lower ASFV DNA levels in tissues. This suggests that the induced immune response does help in eliminating ASFV. This statement is in line with the results observed in recent vaccination trials [12,50], where there was a correlation of antibody response and decrease in the ASFV DNA levels in tissues over time in vaccinated animals. 

## 5. Conclusions

We tested a thirty-five Ad5-ASFV antigens cocktail that could not induce protection against the virulent ASFV Arm07 isolate. Despite the best protective efficacy recorded to date against ASFV being seen in studies with live attenuated vaccine prototypes [11,12,13,14,16,42,43,44,45], continuing investigations based on subunit vaccines is highly interesting from the point of view of finding a safer vaccine. However, the lack of protection obtained in this study along with other similar results from studies that have tested subunit vaccine prototypes against ASFV [19,20,36,37,38,39,40,41] demonstrates that there is still much room for improvement to obtain an effective subunit ASF vaccine. Therefore, further studies are needed to identify novel protective ASFV antigens and an effective in vivo antigen delivery platform capable of priming and expanding protective immunity in a manner similar to attenuated ASFV.

## Figures and Tables

**Table 1:** ASFV antigens encoded by the Ad5 constructs.

Construct ID	ASFV Antigens Expressed by Ad5 Construct
Ad5-ASFV 1	p220.1
Ad5-ASFV 2	p220.2
Ad5-ASFV 3	p72, p15, B602L
Ad5-ASFV 4	p62, p32, p54, EP153R, p10
Ad5-ASFV 5	K205R, A104R, EP402RΔPRR, A151R, B119GL, K196R, CP80R
Ad5-ASFV 6	B438L, R298L, NP419L, K145R
Ad5-ASFV 7	B385R, F334L, CP312R, H108R, F165R
Ad5-ASFV 8	F778R, S273R, MGF100-1L, B66L
Ad5-ASFV 9	NP868R, H339R
Ad5-ASFV 10	I329L, A224L, MGF505-6R, B175L

**Table 2:** Protocol per each group of treated, control and intramuscularly challenged animals.

Group	Sub-Group	Inoculum	Dose	Adjuvant
	T1	Ad5-ASFV	Prime: 10^10^ IFU	BioMize
			Boost: 10^11^ IFU	
Treated	T2	Ad5-ASFV	10^10^ IFU	BioMize
	T3	Ad5-ASFV	Prime: 10^10^ IFU	-
			Boost: 10^11^ IFU	
Control		Ad5-Luc	Prime: 10^10^ IFU	BioMize
			Boost: 10^11^ IFU	
Challenge		Arm07	10 HAD_50_	-

IFU, Infectious focus units; HAD_50_ = Hemadsorption in 50% of infected cultures.

**Table 3:** Clinical signs and scoring specific parameters for ASFV infection in wild boar.

Clinical Sign	Scoring			
	1	2	3	4
**Rectal temperature**	40–40.5 °C	40.6–41 °C	41.1–41.5 °C	>41.5 °C
**Behavior**	Reduced liveliness (stillness)	Get up only to eat and drink (no exploration), head down posture	Get up only when touched	Refusal to get up even when touched
**Body condition**	Pelvic bones only detectable with firm pressure	Pelvic bones obvious	Pelvic bones very prominent	Generalized cachexia
**Skin alterations** *	Localized slight erythema (limbs, ears, etc.)	Generalized erythema	Generalized erythema, multifocal cutaneous petechiae, intense ocular congestion	Extensive cutaneous necrosis/ulceration/subcutaneous hemorrhages
**Ocular/nasal discharge**	Slight discharge	Thick discharge	Thick discharge for more than two sequential days	Bloody discharge
**Joint swelling**	Slight walking difficulties only when getting up	Walking difficulties, a joint swelling	Walking difficulties, generalized joints swelling	Severe joint swelling, impaired walking
**Respiratory symptoms**	Slightly dyspnea	Moderate dyspnea	Severe dyspnea, abdominal breathing	Open-mouth breathing, cough, abdominal breathing
**Digestive symptoms**	Mucus in feces	Diarrhea, vomiting < 24 h	Diarrhea, vomiting > 24 h	Bloody diarrhea, frequent vomiting
**Neurological symptoms**		Stagger gait	Ataxia of the hindquarters	Paralysis of the hindquarters, convulsions

* Pictures illustrating this clinical parameter is provided as Supplementary Material (Figure S1).

**Table 4:** The results of the best-fitting mixed model to explain the ASFV DNA levels in tissues.

	Estimate	Std. Error	t Value	*p*-Value
**(intercept)**	3.12	0.04	84.68	***
**Bone marrow**	−0.13	0.04	−3.57	***
**Brain**	−0.10	0.034	−2.74	**
**Kidney**	−0.12	0.04	−3.25	**
**Liver**	−0.21	0.04	−5.20	***
**First barrier/Lymph nodes**	−0.17	0.02	−7.72	***
**Spleen**	−0.22	0.04	−5.68	***
**Thoracic cavity**	−0.07	0.02	−3.19	**
**Clinical score**	0.01	0.001	5.73	***
**ELISA test**	0.06	0.02	3.48	***
**Days of viremia**	−0.02	0.01	−2.95	**

(P-values: *p* > 0.1 “ns”; *p* < 0.1 “.”; *p* < 0.05 “*”; *p* < 0.01 “**”; *p* < 0.001 “***”). Coefficients of tissues are relative to tissues from abdominal cavity organs. Coefficients of the ELISA test are relative to a negative result.

## Supplementary Materials

The following are available online at https://www.mdpi.com/2076-0817/9/3/171/s1, **Figure S1**: Clinical signs related to skin alterations parameters for clinical scoring in wild boar: localized erythema in ears, eyes and snout (A); intense ocular congestion (B); and generalized erythema (ventral abdomen) (C). **Table S1**: Models fitted to evaluate the ASFV DNA levels in tissues. The best-fitting model is remarked in a square, with the lowest Akaike information criteria (AIC) and Bayesian information criterion (BIC). Marginal (R^2^M) and conditional (R^2^C) squared R are given for each model. **Figure S2**: The predictor effects obtained from the best-fitting mixed model to explain the ASFV DNA levels in tissues. Shaded areas represent the standard error of predictor effects values.
