# Supplementary material for: Adenovirus-vectored African Swine Fever Virus Antigens Cocktail Is Not Protective against Virulent Arm07 Isolate in Eurasian Wild Boar
Source: Pathogens. 2020 Feb 28;9(3):171. doi: 10.3390/pathogens9030171 (PMC7157622; doi:10.3390/pathogens9030171)
Supplement: Supplementary file 1 [file pathogens-09-00171-s001.pdf]

pathogens

MDPI

Supplementary Material

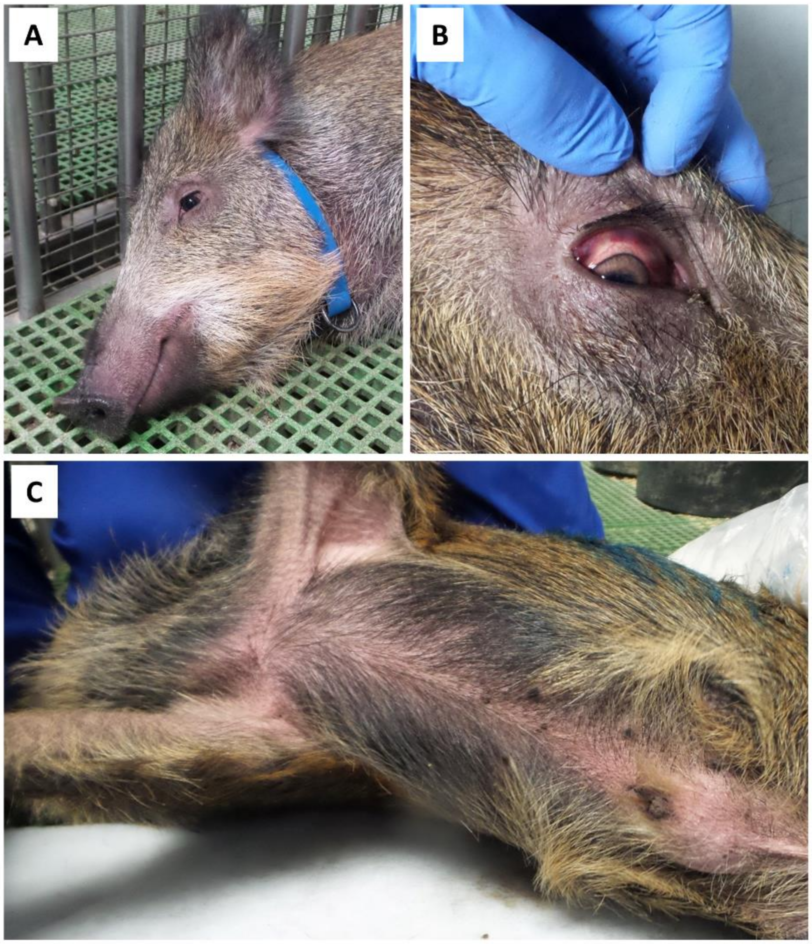

Figure S1. Clinical signs related to skin alterations parameters for clinical scoring in wild boar: (A) localized erythema in ears, eye region and snout, (B) intense ocular congestion and (C) generalized erythema (ventral abdomen).

pathogens

MDPI

Table S1: Models fitted to evaluate the ASFV DNA levels in tissues. The best-fitting model is remarked in a square, with the lowest Akaike information criteria (AIC) and Bayesian information criterion (BIC). Marginal (R^2M) and conditional (R^2C) squared R are given for each model.

	AIC	BIC	R^2M	R^2C
CT value from tissue ~ Tissues + Clinical score + ELISA test + Days of viremia + CT value from last viremia + Temperature + Group of treatment + Survival time (days)	1112	1171	0.36	0.56
CT value from tissue ~ Tissues + Clinical score + ELISA test + Days of viremia + CT value from last viremia + Temperature + Group of treatment	1110	1165	0.37	0.54
CT value from tissue ~ Tissues + Clinical score + ELISA test + Days of viremia + CT value from last viremia + Temperature	1108	1160	0.38	0.53
CT value from tissue ~ Tissues + Clinical score + ELISA test + Days of viremia + CT value from last viremia	1108	1156	0.38	0.52
CT value from tissue ~ Tissues + Clinical score + ELISA test + Days of viremia	1107	1151	0.39	0.51
CT value from tissue ~ Tissues + Clinical score + ELISA test	1113	1154	0.37	0.51
CT value from tissue ~ Tissues + Clinical score	1122	1159	0.53	0.50
CT value from tissue ~ Tissues	1118	1152	0.24	0.50

pathogens

MDPI

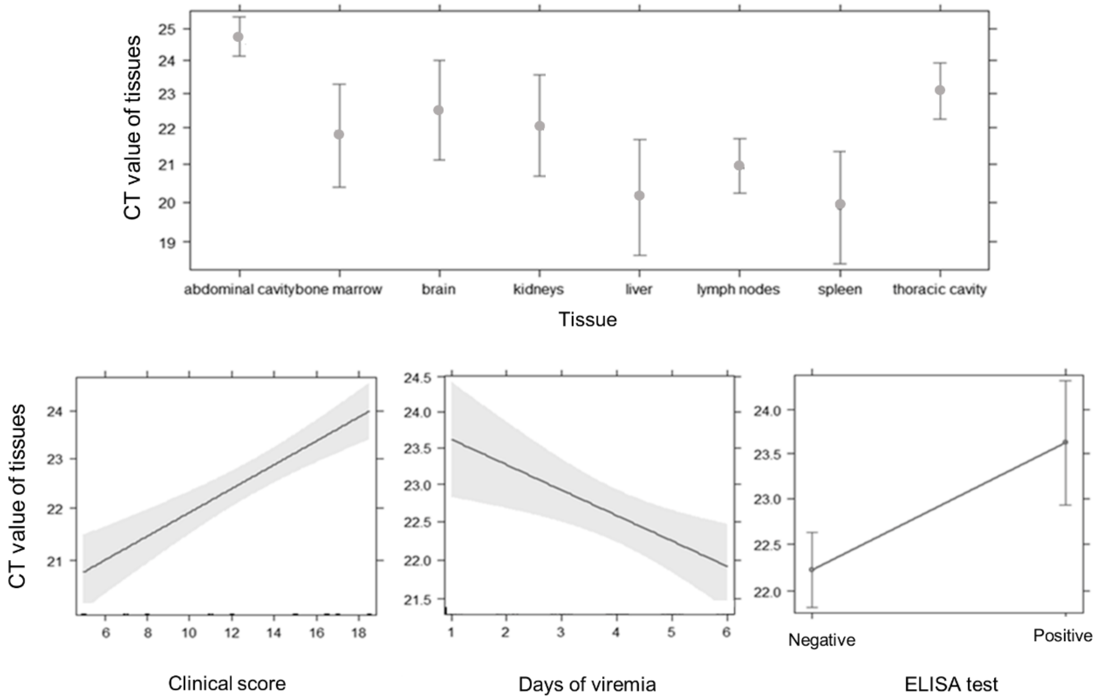

Figure S2. The predictor effects obtained from the best-fitting mixed model to explain the ASFV DNA levels in tissues. Shaded areas represent the standard error of predictor effects values.
